# An Investigation of the Behavior of Solvent based Polycaprolactone ink for Material Jetting

**DOI:** 10.1038/srep20852

**Published:** 2016-02-12

**Authors:** Yinfeng He, Ricky D. Wildman, Chris J. Tuck, Steven D. R. Christie, Steven Edmondson

**Affiliations:** 1 Additive Manufacturing and 3D Printing Research Group, University of Nottingham, Nottingham, NG9 2RD, UK; 2 Department of Chemistry, Loughborough University, LE11 3TU, UK; 3 School of Materials, The University of Manchester,M13 9PL, UK.

## Abstract

An initial study of processing bioresorbable polycaprolactone (PCL) through material jetting was conducted using a Fujifilm Dimatix DMP-2830 material printer. The aim of this work was to investigate a potential solvent based method of jetting polycaprolactone. Several solvents were used to prepare a PCL solvent based ink and 1, 4-dioxane was chosen with the consideration of both solubility and safety. The morphology of PCL formed under different substrate temperatures, droplet spacings were investigated. Multi-layer PCL structures were printed and characterized. This work shows that biodegradable polycaprolactone can be processed through material jetting.

There is an increasing need for the development of new manufacturing methods to produce bioresorbable products within the medical implant industry. Two of the main functions for such a product are to provide structural support to aid tissue recovery and to serve as the platform for drug dissolution and delivery. Currently, resorbable objects are generally made using standard industrial processes, but there is a growing need to produce customized products that are created to match the needs^1^. A promising route to such products is additive manufacturing (AM), commonly referred to as 3D printing (3DP). Additive manufacturing is a suite of processes that involve the addition of material on a layer by layer basis^2^,^3^. AM includes several different technologies, for example: Fused Deposition Modeling (FDM) is an extrusion based technique which is limited in build speed and accuracy^2^; Powder Bed Fusion (PBF) is a process to fuse powder based materials together, which has wide range of applicable materials though the resolution relies on the particle and laser interaction properties^3^; Material Jetting is a drop-on-demand inkjet method with a high spatial resolution that deposits one drop of ink at a time by jetting and then a reaction or evaporation is triggered by an external energy source (e.g. ultra-violet light, heating) to form a solid structure^3^,^4^.

A number of materials have been identified as having significant potential for bioresorbable applications. These include poly(lactic) acid, polyvinyl alcohol and polycaprolactone (PCL), which have different properties and behavior, often making them complementary^5^. The last of these, PCL, is a semi-crystalline and hydrophobic biodegradable polymer, which resists random hydrolytic chain scission of the ester groups and as a consequence degrades over a relatively long degradation period compared to other bioresorbable polymers. This represents a useful starting point for 3D printing of bioresorbable products with long term applications, particularly those applications such as bone fracture implants where structural performance is required for many weeks^6^. PCL has previously been investigated for AM applications^7^,^8^,^9^,^10^, most notably using Fused Deposition Modelling (FDM). However, this process is limited in resolution, particularly when one is interested in co-processing other materials, it is usually limited to one, sometimes two, materials. It also has limited scalability, which is a requirement for any manufacturing process. Material jetting opens up a vista where such multi-material deposition becomes possible. In order to jet PCL, it is necessary to prepare it into a state where the key rheological parameters fall within those that allow printing.

Teichler *et al*.^11^ and Lee *et al*.^12^ were able to print polymer films by dissolving target polymers into appropriate solvents, determining in the process that droplet spacing and substrate temperature were critical to the film quality. In another study, Shin *et al*.^13^ determined the form of the pressure waveform required for printing low viscosity inks including water and water based mixtures. Evidence from the literature also shows that PCL can be effectively dissolved in solvents such as chloroform and that the pharmaceutical industry has established solvents that may be exploited, including ethanol, ethyl acetate, chloroform and 1, 4-dioxane^14^,^15^,^16^. These studies have shown the usage of PCL in biomedical applications, whilst also establishing the potential for material jetting based AM in the manufacture of resorbable objects formed from PCL. In this paper, the aim is to establish the basis by which PCL can be printed, determining appropriate solvents that show evidence of falling within safety guidelines, and determining the set of printing parameters that will afford jetting PCL.

## Results and Discussion

### Solubility Test

The solubility of PCL granules in a range of solvents including 1, 4-dioxane, chloroform, ethyl acetate and ethanol were assessed through observing the manner of dissolution. PCL was found to be fully dissolved in 1, 4-dioxane and chloroform after 24 hours. However, when in ethyl acetate, debris was observed to peel off from the granules’ outer surface, but no significant dissolution was observed. Similarly, no dissolution was observed in ethanol, but in this case no debris was formed either. As a result, chloroform and 1, 4-dioxane were determined as potential solvents for ink preparation. To further narrow down the choice of solvent, it was noted that the permissible daily exposure limit of 1, 4-dioxane is 380 ppm/g which is over six times the allowable limit for for chloroform (60 ppm/g)^16^. This led to 1, 4-dioxane being chosen as the solvent most likely to be appropriate for formulation of PCL based inks.

### Ejectability Assessment

**Figure 1:**
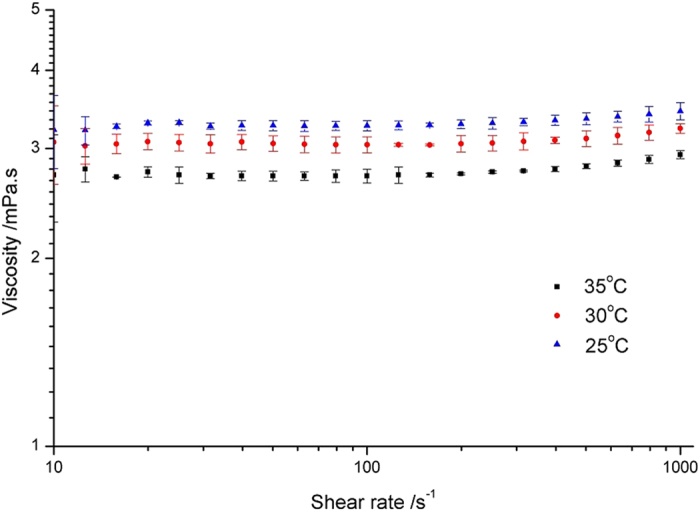
Viscosity of 5 wt% PCL solvent ink tested from 25 °C to 35 °C with shear rate range between 10 s^−1^ to 1000 s^−1^. Data are expressed as the mean ± standard deviation, n = 3.

**Figure 2:**
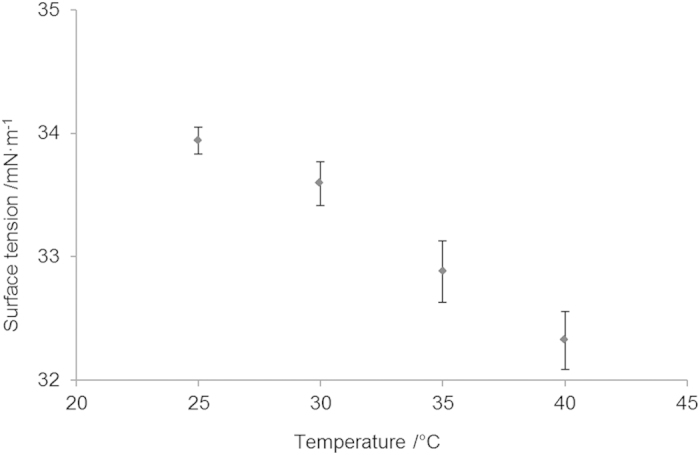
Surface tension of 5 wt% PCL solvent ink tested between 25 °C and 35 °C. Data are expressed as the mean ± standard deviation, n = 4.

The results of the shear rate sweep are shown in Fig. 1. It can be observed that the viscosity was 3.29 ± 0.05 mPa.s at 25 °C which then dropped to 2.75 ± 0.06 mPa.s when the plate temperature was increased to 35 °C. The surface tension of the ink was also measured as a function of temperature (Fig. 2). It was found that the surface tension was around 34 mN/m at 25 °C which then reduced when the temperature was increased. This is consistent with the Eötvös relationship of linearly reducing surface tension with temperature^17^. The remaining relevant properties to estimate ejectability were also determined and are shown in Table 1. The printing indicator *Z* at different temperatures was calculated. The calculated Z value shows the ink was inside the ejectable range between 25 °C and 35 °C. However during actual jetting, it was found that as the printing temperature increased, the nozzle became more unstable and clogging occurred frequently due to evaporation of ink at the nozzle, suggesting other conditions, such as vapour pressure, must be considered when deciding on ‘ejectability’. For reliability reasons, in this study, 25 °C was used as the print temperature.

### Droplet formation

**Figure 3:**
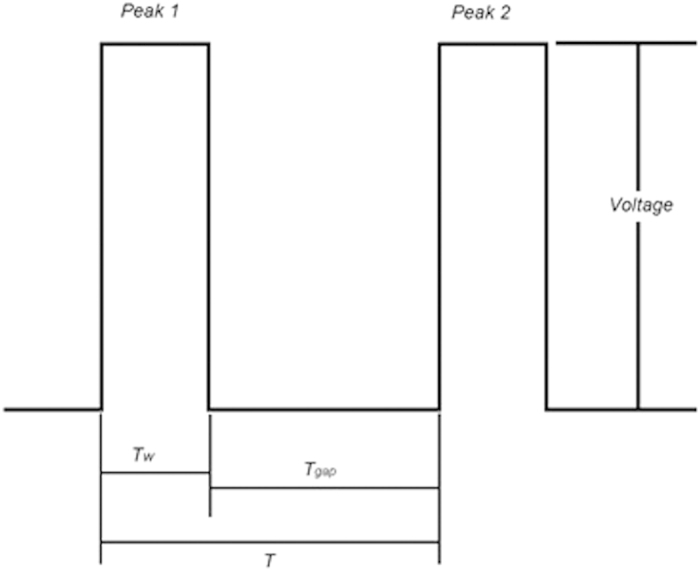
Double peak waveform used in this research, where *T* is time gap, *T_w_* is peak width, *T*_*gap*_ is peak gap.

**Figure 4:**
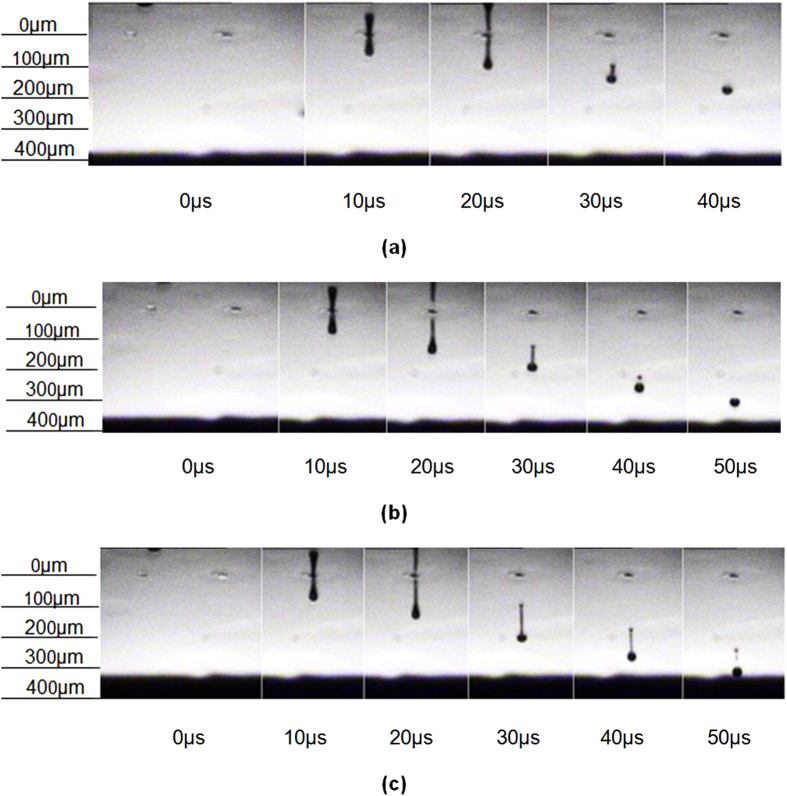
Three types of different droplet formation states: (a) single droplet without satellite, (b) satellite with recombination and (c) satellite without recombination.

**Figure 5:**
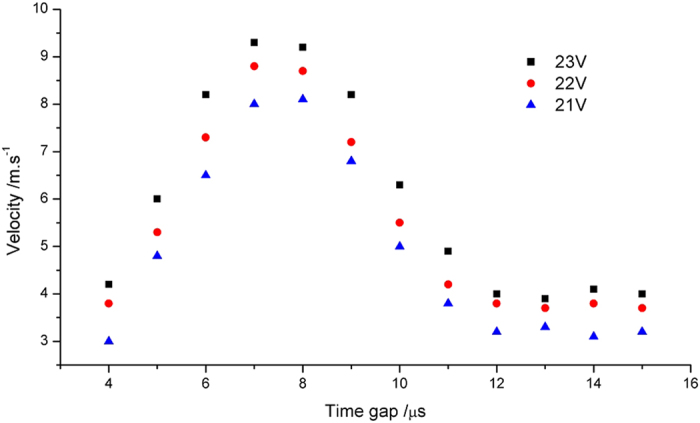
Droplet Velocity under different time gaps and printing voltages.

At this stage, the ‘satellite effect’ was observed - where small droplets are formed from the break-up of the primary droplet tail. This effect can be minimized by manipulation of the printing waveform, which has been shown by Shin *et al*.^13^ and Dong *et al*.^18^,^19^. Dong *et al*.^18^,^19^ also explained the mechanism which caused satellite formation. A double peak waveform (Fig. 3) was chosen for the initial PCL solvent based ink and this has been shown to reduce the formation of satellites. In this study the time gap (*T*) between pulses was varied between 4 and 15 *μ*s, whilst the width of the peaks was fixed at 3 *μ*s. The printing voltage was an additional variable that affects the pressure pulse height. Three kinds of droplet formation states were observed when changing the printing voltage and time gap (Fig. 4). The situations in Fig. 4(a,b) were defined as acceptable in this study as only one primary droplet was formed before reaching the substrate. Dong *et al*.^18^,^19^ studied these effects in detail and they concluded that whether satellite without recombination happened or not depended on the thread length and primary droplet speed when the thread broke up. They demonstrated that the retreating speed of the thread tail was almost constant for an ink while the primary droplet speed was related to printing voltage and waveform. When the retreating speed was lower than the primary droplet speed, satellite without recombination will appear. Therefore, the satellite effect can be minimized by adjusting the front droplet speed. Figure 5 shows how the time gap (*T*) between the starting points of two peaks and printing voltage affected the droplet velocity, which reached a maximum at a time gap of around 7 *μ*s. Increasing the printing voltage increased the velocity of the primary droplet for any given time gap.

The primary droplet speed variation by time gap can be explained, in some cases, by investigating the pressure waves being transmitted and reflected within the ejection chamber. For example, Kwon *et al*.^20^ indicated that the pressure waves will superimpose and act to dissipate the pressure wave when the time gap (*T*) between the start of the second peak and that of the first peak, is either 2*L*/*c* or 6*L*/*c* (where *L* is the channel length and *c* is the speed of sound inside the ink). This can be expressed in terms of the peak gap, *T*_*gap*_, and peak width, *T*_*w*_, such that *T* = *T*_*gap*_ + *T*_*w*_. This gives some indication of the bounds on the separation of the two peaks. The channel length in the case of the Dimatix cartridge is 3 mm and the speed of sound in the ink can be estimated from the speed of sound in 1, 4-dioxane with 5 wt% polystyrene, which is 1360 m/s^21^. By plotting the velocity of the drop versus *T*, it was found that the minimum velocities occurred at approximately 4 *μ*s and 12 *μ*s (Fig. 5), matching closely the predictions from Kwon *et al*. Going beyond these values for T, one would see that the two voltage peaks will join together if we reduced its value, and when further increasing beyond 12 *μ*s, no substantial velocity changes were observed. 

For our PCL based system, therefore, Kwon *et al*.’s analysis reasonably predicts the bounds for any optimization of the droplet formation, providing a starting point for obtaining printing parameters.

### Droplet Deposition

**Figure 6:**
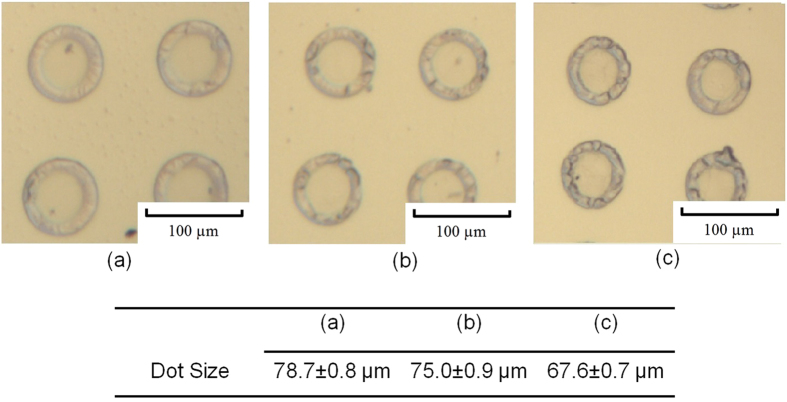
Optical microscopy images of solidified 5 wt% PCL solvent ink printed at different substrate temperatures (a) 25 °C (b) 30 °C (c) 35 °C. Data are expressed as the mean ± standard deviation, n = 3.

The effect of changing substrate temperature on the deposited droplet size was investigated. Images of the solidified PCL ink are shown in Fig. 6. It can be seen that solidified PCL tends to concentrate near the outer perimeter of the droplet under all used printing conditions. This phenomenon is commonly known as the ’Coffee Ring’ effect and often occurs with suspension based inks^12^,^22^,^23^. As evaporation is the key step to removing solvent and allowing the PCL to solidify, the effect of the temperature on the morphology of the solidified deposits was investigated by varying the substrate temperature and then observing the size and condition of the deposits using an optical microscope. It was observed that the sizes of the solidified droplet slightly reduced as the substrate temperature was increased, an affect that may be attributed to changes in the wettability and surface tension of the materials with temperature.

### Line and Film Formation

The formation of ink lines and films from individual ink droplets have been studied previously with other types of inks^11^,^24^,^25^,^26^, in which the substrate temperature and the droplet spacing were investigated to examine their influences on the printed pattern morphology and used to decide a suitable printing and solidification condition. Gao *et al*.^26^ and Stringer *et al*.^24^ also suggested a way to predict the printed line width through contact angle, droplet size and droplet spacing. Similar experiments were carried out in this paper to decide a suitable combination of substrate temperature and droplet spacing for jetting 1, 4-dioxane based PCL solvent ink.

Three different droplet spacings (20 *μ*m, 40 *μ*m and 60 *μ*m) were chosen to investigate how droplet overlap influences the morphology of printed lines. The effect of different substrate temperatures was also examined since it has already been observed that variation in temperature can lead to changes in deposit diameter, and which should have a knock on effect on the line formation too.

**Figure 7:**
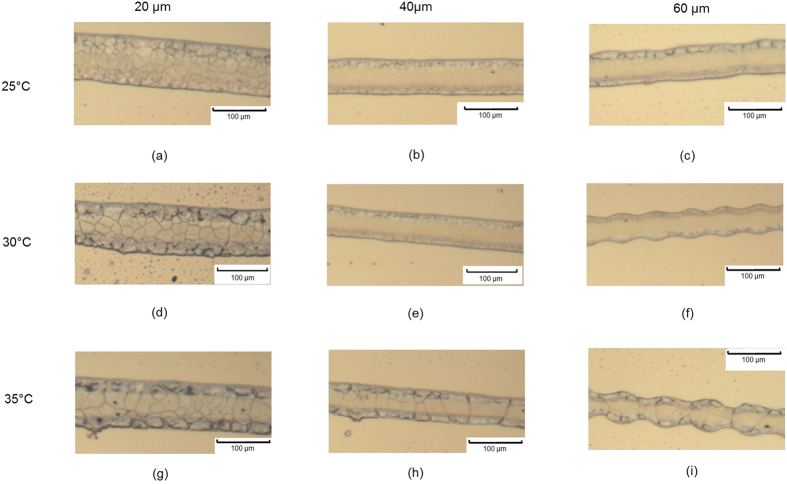
Optical microscopy results of line formation test for PCL solvent ink printed with different droplet spacing and substrate temperatures.

**Figure 8:**
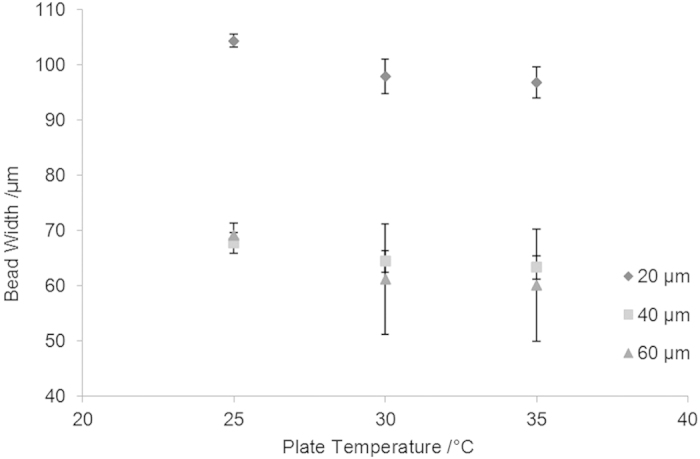
A plot of solidified PCL line width against plate temperature with different droplet spacing: 20 *μ*m, 30 *μ*m and 40 *μ*m. Data are expressed as the mean ± standard deviation, n = 3.

Microscope images of the lines are shown in Fig. 7 with the average line widths shown in Fig. 8. It can be observed that when droplet spacing was 20 *μ*m, considerably wider tracks appeared compared with those seen when 40 *μ*m and 60 *μ*m droplet spacing was employed. The reduced droplet spacing resulted in highly overlapped droplets and a large volume of ink per unit area. It is likely that the triple line of the deposited droplets was unable to support such a large amount of ink and moved outwards in response^25^. Additionally, the printed track widths were seen to be dependent upon the substrate temperature.

Table 2 shows the measured line widths as a function of temperature and droplet spacing. This was compared with the predictions given by Gao *et al*.^26^ and Stringer *et al*.^24^. The comparison is reasonable, with differences typically less than 10%. This suggests that this can be a useful guide for manufacturing, allowing the user to predict the line spacing required to achieve reasonable coverage of the substrate, at least within the constraint that the deposited drops were within the range of separation that allowed them to merge and form a cylindrical cap.

**Figure 9:**
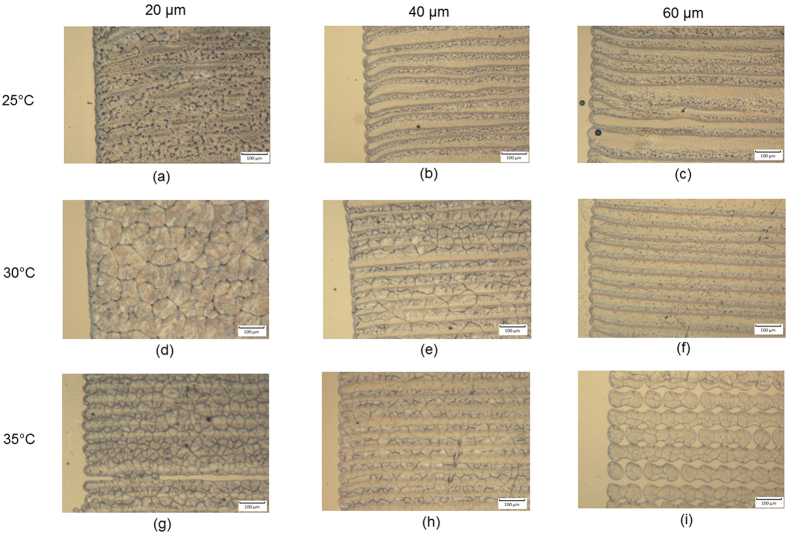
Optical microscopy pictures of printed PCL solvent film with droplet spacing equals to 20 *μ*m, 40 *μ*m, 60 *μ*m respectively and substrate temperatures of 25 °C, 30 °C and 35 °C.

The PCL films were printed with 20 *μ*m , 40 *μ*m and 60 *μ*m droplet spacing with substrate temperatures of 25 °C, 30 °C and 35 °C respectively to investigate the effect of droplet spacing as well as the substrate temperature on PCL film formation. Figure 9 shows the patterns that were formed when jetting was performed under a range of parameters, most notably showing periodic regular structures that appear to correspond to the regularity of the tracks.

It can be seen that for films printed with 20 *μ*m droplet spacing, no substantial waviness was observed until the substrate temperature reached 35 °C. This was likely caused by the competition between ink evaporation and merging. When the ink was printed with a 20 *μ*m droplet spacing (70% overlapping) at 25 °C, the printed ink dissolved and merged with previously unsolidified ink. As the substrate temperature increased, the solidification speed also increased (due to evaporation). When a printed row of ink can complete the solidification process before the next row of ink is deposited, the boundary interface becomes obvious. (Fig. 9(g)).

When the droplet spacing was fixed to either 40 *μ*m or 60 *μ*m, the regular waviness was seen regardless of the temperature. This was potentially because that when the film samples were printed with 40 *μ*m and 60 *μ*m droplet spacing, the ink volume within a unit area reduced by 74% and 86.5% respectively compared to those printed with 20 *μ*m droplet spacing. This implies that less solvent needs to be evaporated leading to a reduction in solidification time and the formation of strong interfacial features and a regular waviness. When substrate temperatures reached 35 °C, nozzle failure was observed and printing became unstable. This was due to the nozzle plate being heated up by the substrate during jetting, which accelerated the evaporation of the ink and caused nozzle blockage. A solution to this may be to increase the stand off height between the printhead and the substrate, but this may come with the cost of reduced precision as the drops will be influenced by small air currents within the chamber.

The quality of printed films under different printing parameters were characterized by measuring the Mean Roughness value (*R*_*a*_) through a Bruker ContourGT-I microscope with a spot area of 500 *μ*m × 500 *μ*m. From Table 3, it can be concluded that a 40*μ*m droplet spacing with 30 °C and 35 °C substrate showed lower *R*_*a*_. However, as mention before that when substrate temperature reached 35 °C, nozzle failure was observed frequently. Therefore, 30 °C and 40 *μ*m droplet spacing were chosen as the conditions for multi-layer printing.

### Multi-Layer Jetting

**Figure 10:**
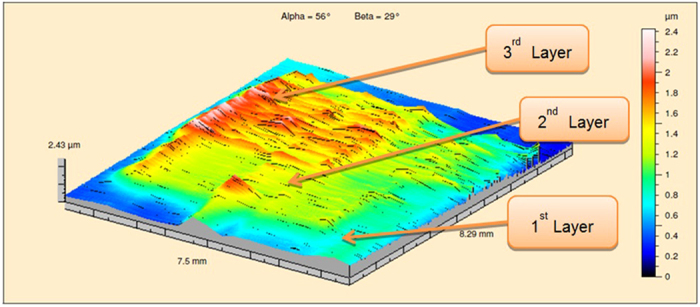
Surface profile of a stepped PCL square sample printed with polycaprolactone solvent based ink (5 wt%) with 30 °C substrate temperature and 40 *μ*m droplet spacing.

**Figure 11:**
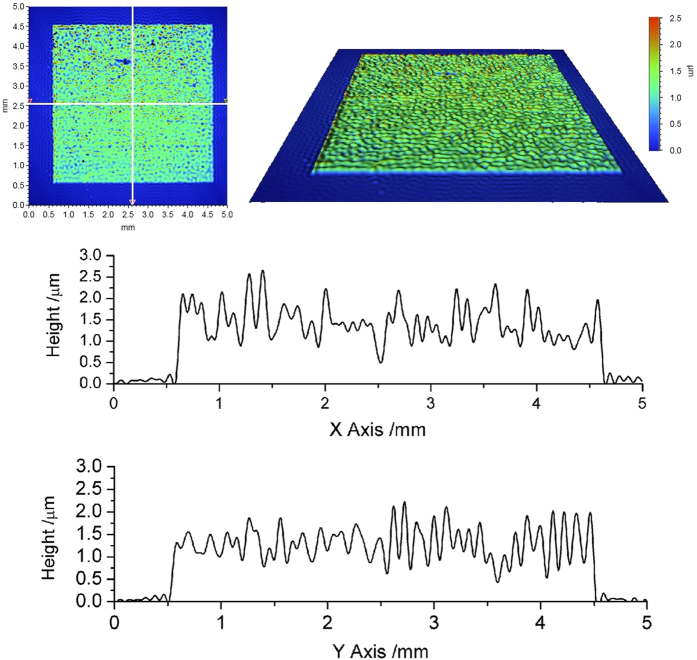
Surface profile of a 3 layers PCL square sample printed with polycaprolactone solvent based ink (5 wt%) with 30 °C substrate temperature and 40 *μ*m droplet spacing.

Multi-layer jetting demonstrated the possibility of creating layer based structures and formation of a 3D object. Multilayered samples of PCL 5 wt% solvent ink were printed with 40 *μ*m droplet spacing and a substrate temperature of 30 °C. The multilayer samples were characterized using a probe based Talysurf 2000. Figure 10 shows the surface profiling result of a printed PCL sample with three different sizes of squares (6 mm × 6 mm for the first layer, 4 mm × 4 mm for the second layer and 2 mm × 2 mm for the third layer). From Fig. 10, it can be observed that the thickness of the first layer was around 0.7*μ*m while the second layer was about 1.4 *μ*m and the third layer around 1.9 *μ*m. Another 4 mm × 4 mm square sample was printed, which consisted of 3 layers of PCL 5 wt% solvent ink and was characterized by Bruker ContourGT-I microscope (Fig. 11). The profiling data showed that the maximum height of the square sample was around 2 *μ*m. The average thickness of the square was around 1.5*μ*m. The mean surface roughness increased with increasing number of layers (*R*_*a*_ = 0.86 *μ*m), perhaps reflecting that multi-layer printing may amplify heterogeneities as the film was built up.

## Methods

Our methodology will be first to establish the appropriate materials for generating a printable ink solution that will result in bulk PCL being created in the desired formation. The quality of the inks will be determined by considering the major influences on how the ink was printed and how it affects measures such as the shape and appearance of deposited material. Each measurement was repeated three times to get the average and standard deviations.

### Ink Preparation

Polycaprolactone (Sigma-Aldrich Mw 10 K) was used as received. The solvents used in this study were all purchased from Sigma-Aldrich including Chloroform (99%, PCR reagent, contains amylenes as a stabilizer), ethanol (puriss. p.a., absolute, 99.8% (GC)), ethyl acetate (anhydrous, 99.8%) and 1, 4-dioxane (anhydrous 99.8%). Polycaprolactone granules were dissolved into each of the solvents at a 5 wt% concentration level and then allowed to settle for 24 hours at room temperature. They were then stirred using an IKA RCT Basic IKAMAG Magnetic Stirrer (with temperature controller) at 800 rpm at room temperature for 10 minutes to improve the dispersion of the dissolved PCL. Microscope glass slides (75 mm × 25 mm × 1.0 mm) were used as the printing substrate. Before printing, the slides were soaked into 2-propanol (99.9%, Fisher Scientific) and ultrasonicated (Branson Ultrasonic Bath Model 1210) for 5 minutes. Then they were rinsed with deionized water and dried in air. The inks were passed through a nylon 5.0 *μ*m syringe filter (Cole-Parmer) before being injected into a printhead cartridge to remove any particulates that might block the nozzles.

### Ejectability Assessment

A factor for characterizing ‘ejectability’ or interface rupture of droplet formation for an ink is the *Z* number^2^,^27^. This is given by:


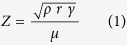


where *ρ* is the density, *r* is the characteristic length (in this case the nozzle diameter), *γ* is the surface tension of the fluid and *μ* is the viscosity of ink. Lower *Z* value means viscous forces become more important and therefore more energy is required to produce an identical droplet^27^,^28^.

A value of *Z* between 1 and 10 for a ink tends to suggest that ejecting this ink through drop-on-demand printing is possible^2^,^28^. The viscosity of the formulated ink was assessed using a 40 mm parallel plate geometry (Malvern Kinexus Pro) with a shear rate sweep between 10 *s*^−1^ to 1000 *s*^−1^ within a temperature range of 25 °C to 35 °C and a 5 °C interval. The plate gap was 150 *μ*m. The surface tension of the droplet was measured by pendant drop shape analysis (Kruss DSA100S).

### Inkjet Deposition Assessments

Following ejectability assessments, PCL ink was printed using a Dimatix DMP 2830 (FujiFilm) with a printhead consisting of 16 nozzles and nozzle size of 21 *μ*m. About 2 ml of ink was injected into the cartridge prior to printing. The droplet formation of the PCL solvent ink was observed with a double pulse waveform. The effects of different printing parameters including substrate temperature and droplet spacing were investigated to study their influences on the printing quality. Optical microscopy pictures of printed samples were taken using a Reichert-Jung MEF3. The printed PCL film quality was characterized using a Bruker Contour GT-I and Talysurf 2000.

## Conclusion

Material jetting was used as a processing method to print polycaprolactone. It was found the temperature can play an important role in the quality of the printing. At the droplet deposition stage, it was found that PCL tended to concentrate at an outer ring after solidification, though increasing the temperature could reduce the size of the deposit. During line formation tests, higher substrate temperature and larger droplet spacing both caused printed PCL tracks to decrease in width. Higher substrate temperature made the boundary interfaces between different printing tracks become clearer and more obvious. The observations demonstrated it was possible to optimize temperature and drop spacing to improve film quality. The chosen solvent, 1,4-dioxane, enabled the printing of PCL, whilst also enabling rapid evaporation of solvent and the creation of a solid film. Multi-layer deposition was achieved, suggesting the manufacture of 3D objects from PCL through Material Jetting is a possible route forward.

## Additional Information

**How to cite this article**: He, Y. *et al*. An Investigation of the Behavior of Solvent based Polycaprolactone ink for Material Jetting. *Sci. Rep*. **6**, 20852; doi: 10.1038/srep20852 (2016).

## Figures and Tables

**Table 1:** Physical properties and printing indicator of PCL solvent ink at a temperature of 25 °C, 30 °C and 35 °C.

Temperature (°C)	Nozzle Diameter *(μ*m)	Density (*g*/*cm*^3^)	Viscosity (mPa.s)	Surface Tension (mN/m)	Z
25	21	1.03	3.29	33.9	8.21
30	21	1.03	3.07	33.6	8.98
35	21	1.03	2.75	32.9	9.88

Viscosity and surface tension data are expressed as the mean, n = 3–4.

**Table 2:** Comparison of theoretical printing line width and actual line width with different droplet spacing and platform temperatures.

Average Line Width (*μ*m)						
Substrate Temperature	Droplet Spacing (*μ*m)					
	20 *μ*m		40 *μ*m		60 *μ*m	
	Theoretical	Actual	Theoretical	Actual	Theoretical	Actual
25 °C	107.06	104.3 ± 1.2	75.70	69.7 ± 1.9	61.81	69.1 ± 2.2
30 °C	99.58	97.9 ± 3.1	70.41	64.4 ± 2.0	57.49	61.2 ± 10.0
35 °C	85.16	96.8 ± 2.8	60.22	63.3 ± 2.1	49.17	60.1 ± 10.2

Data are expressed as the mean ± standard deviation, n = 4.

**Table 3:** The mean roughness of printed PCL films.

The mean roughness value (*R*_*a*_) (*μ*m)			
Substrate Temperature	Droplet Spacing (*μ*m)		
	20 *μ*m	40 *μ*m	60 *μ*m
25 °C	0.26 ± 0.03	0.29 ± 0.06	0.25 ± 0.04
30 °C	0.21 ± 0.03	0.19 ± 0.02	0.21 ± 0.03
35 °C	0.24 ± 0.05	0.19 ± 0.03	N/A

Data are expressed as the mean ± standard deviation, n = 4.
